# From bioadsorption to biotransformation: a hypothesis for mercury bioremediation using genetically modified microalgae

**DOI:** 10.3389/fmicb.2026.1724533

**Published:** 2026-01-21

**Authors:** Chang-ye Hui

**Affiliations:** Shenzhen Prevention and Treatment Center for Occupational Diseases, Shenzhen, China

**Keywords:** genetically modified microalgae, intracellular and extracellular detoxification, MerA and MerB enzymes, mercury bioremediation, mercury detoxification

## 1 Introduction

Environmental microorganisms have evolved specific resistance operons in response to diverse heavy metal stresses. Among these, the MerR family of metalloregulators, named after the Hg(II)-responsive MerR, stands out. This family includes MerR for mercury resistance, cadmium resistance regulator (CadR) for cadmium resistance (Guo et al., 2025a), and lead resistance regulator (PbrR) for lead resistance (Jung and Lee, 2019). These MerR family metalloregulators are renowned for specifically recognizing their target metals and activating the expression of downstream resistance genes. This specificity in metal recognition allows researchers to exploit these metalloregulators for targeted bioremediation strategies. Engineered bacteria expressing MerR intracellularly have been shown to accumulate and detoxify mercury. For example, Chen et al. introduced a synthetically engineered *Escherichia coli* biosensor, MerR, with a mercury binding function into the intestines of mice, which significantly enhanced Hg^2+^ resistance and promoted the excretion of oral Hg^2+^ with feces, thereby reducing the concentration of mercury in the circulatory system and organs (Chen et al., 2023).

Alternatively, through surface display technology, these metalloregulators can be presented on the outer membrane of bacteria, facilitating the capture and removal of target heavy metals, such as Hg(II), Cd(II), Pb(II), and As(III), from contaminated environments. These research findings have been summarized in our previous reviews (Hui et al., 2021, 2023b, 2024a,b; Hui, 2025).

## 2 Innovative approach to mercury bioremediation using *Chlamydomonas reinhardtii*

The article titled “Surface displayed MerR increases mercury accumulation by green microalga *Chlamydomonas reinhardtii*,” published in 2024 in the journal Environment International (volume 189), presents an innovative approach to enhancing mercury accumulation using the green microalga *Chlamydomonas reinhardtii* (*C. reinhardtii*). The researchers engineered the algal cells to display the metalloregulatory protein MerR on their surface, which is known for its high affinity and selectivity for Hg^2+^ (Ou et al., 2025). This novel cell surface engineering strategy significantly increased the accumulation of Hg^2+^ by the algal cells compared to wild-type strains, demonstrating a promising method for mercury bioremediation (Chokshi et al., 2024). Notably, the study utilized *C. reinhardtii*, a well-established aquatic model organism, to validate the effectiveness of the engineered surface adsorbent for capturing Hg^2+^, highlighting the potential of this green microalga in environmental mercury removal applications.

## 3 Overlooking methylmercury: a critical gap in the study of mercury bioremediation by *Chlamydomonas reinhardtii*

Despite the innovative approach and promising results presented in the study on the surface display of MerR in *C. reinhardtii*, there are notable limitations, particularly in its failure to address the critical issue of MeHg. The study's focus on inorganic Hg^2+^ is an oversight. MeHg represents a more toxic and bioaccumulative form of mercury, posing greater risks to both aquatic ecosystems and human health (Gionfriddo et al., 2016; Guo et al., 2025b). The omission of MeHg is particularly concerning given its propensity for biomagnification in aquatic food chains. MeHg can accumulate to high concentrations in top predators such as large fish, which are often consumed by humans, posing a significant health risk (Cai et al., 2024). By neglecting to discuss MeHg, the study fails to address the full spectrum of mercury toxicity, which is essential for developing comprehensive bioremediation strategies.

The study's approach of displaying MerR on the surface of *C. reinhardtii* is designed to target inorganic Hg^2+^, leveraging the metalloregulatory protein MerR's high affinity for this form of mercury (Tulin et al., 2024; Liu et al., 2025). However, it is essential to acknowledge that the exclusive focus on Hg^2+^ is somewhat inevitable, given the inherent specificity of MerR, which responds only to inorganic Hg^2+^. Thus, the study's strategy is limited by the biochemical properties of the MerR protein itself (Tulin et al., 2024). Nevertheless, this strategy overlooks the broader context of mercury bioremediation, where both inorganic and organic forms of mercury coexist in the environment (Ray et al., 2025). The MerR protein specifically responds to Hg^2+^, but in natural settings, mercury often exists in various forms, including the highly toxic MeHg. In bacterial mercury resistance, the MerR protein is part of a broader *mer* operon that includes MerB, an enzyme capable of cleaving the carbon-mercury bond in organic mercury compounds, converting them into inorganic Hg^2+^. This inorganic form can then be reduced to elemental mercury (Hg^o^) by MerA, another enzyme in the *mer* operon, thereby detoxifying the mercury (Hui et al., 2024b). While the study's approach is understandable given the specificity of MerR, it is crucial not to overlook the significant threat posed by organic mercury in aquatic ecosystems. Organic mercury, particularly MeHg, is more readily taken up by organisms due to its ability to cross cell membranes, leading to higher toxicity and greater bioaccumulation potential (Ray et al., 2025).

Moreover, it is worth noting that the focus on inorganic Hg^2+^ is not unique to this study. Previous research efforts in the field have predominantly concentrated on the bioremediation of Hg^2+^, with few studies addressing the more toxic and bioaccumulative MeHg (Yang et al., 2023).

Given the significant limitations of existing mercury bioremediation strategies, particularly the neglect of MeHg, there is a pressing need for innovative approaches that address both inorganic and organic forms of mercury. This necessity has inspired the development of transformative strategies that leverage the unique capabilities of genetically modified microalgae. Unlike conventional strategies that exclusively target inorganic Hg^2+^, our proposed GMO strategy uniquely addresses the most toxic and bioaccumulative form of mercury by co-expressing MerB and MerA in microalgae, thereby enabling direct demethylation of MeHg—a capability that existing Hg^2+^-focused methods fundamentally lack.

## 4 Transformative strategies in mercury bioremediation: from intracellular to extracellular detoxification using genetically modified microalgae

### 4.1 Mercury geochemical cycle and bioremediation

**Figure 1:**
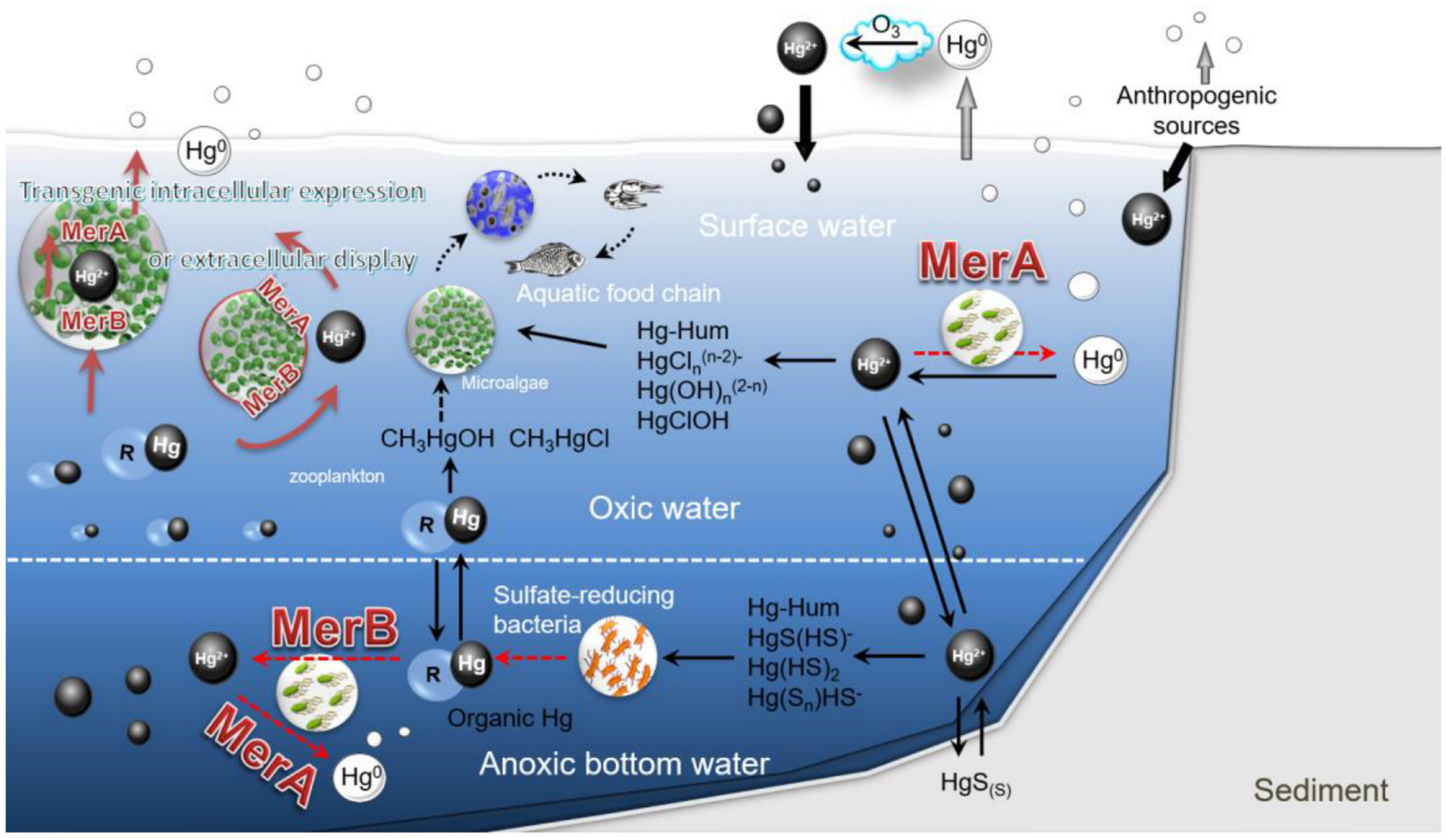
Conceptual framework of mercury bioremediation using genetically modified microalgae for intracellular and extracellular detoxification. This figure combines established geochemical pathways (black solid arrows) with hypothesized engineered microalgal functions (red solid arrows). Established processes include natural methylation and demethylation, as well as Hg^o^ volatilization, by native microbial communities. Hypothetical steps requiring experimental validation include: (1) microalgal uptake of MeHg via heterologous transporters; (2) intracellular demethylation by MerB; (3) surface-displayed MerA/MerB activity; and (4) quantitative Hg^o^ flux rates from engineered cells. Unknown parameters (e.g., kinetic rates, enzyme stability, and HGT frequency) will be investigated further. R, alkyl group, Hum, humic substances, HgS(s), solid mercury sulfide, HgS(n), nanoparticulate mercury sulfide.

Mercury undergoes a complex environmental geochemical cycle, involving various forms such as Hg^o^, Hg^2+^, and MeHg (Barkay et al., 2003). These forms interconvert through biotic and abiotic processes, with significant implications for environmental and human health (Figure 1). The *mer* operon plays a crucial role in mercury detoxification by converting Hg^2+^ and MeHg into volatile Hg^o^ (Hui et al., 2024b). However, this transformation represents a compartmental transfer rather than elimination, as volatilized Hg^o^ can undergo atmospheric transport and re-oxidation, redistributing mercury rather than removing it from the biosphere. Despite the importance of MeHg in aquatic ecosystems due to its high toxicity and bioaccumulation potential, current biological remediation strategies primarily focus on the adsorption and reduction of Hg^2+^.

### 4.2 Case studies in mercury bioremediation

In a recent comment published in *Lab Animal*, we discussed two notable studies that highlight the potential of engineered organisms for mercury bioremediation (Guo and Hui, 2025). Tepper et al. (2025) demonstrated the potential of transgenic animals expressing MerA and MerB enzymes to mitigate mercury toxicity. Specifically, they engineered *Drosophila melanogaster* and *Danio rerio* to express these enzymes, which can demethylate MeHg and reduce Hg^2+^ to Hg^o^ (Tepper et al., 2025). Similarly, Yu et al. (2025) introduced an engineered gut bacterium, *Bacteroides thetaiotaomicron*, expressing MerA and MerB, which significantly reduced MeHg levels in the tissues of pregnant mice fed a diet containing MeHg (Yu et al., 2025). These studies underscore the potential of engineered organisms to transform toxic mercury compounds into less harmful forms, thereby mitigating environmental and health risks.

Building on these advancements, the field of phytoremediation has also seen significant progress. Various plant species, including *Arabidopsis thaliana*, tobacco, poplar, and rice, have been genetically modified to express *merA* and *merB* genes, enabling them to accumulate and detoxify mercury in contaminated soils. These transgenic plants have shown enhanced tolerance to high levels of mercury, with some accumulating Hg in their roots at concentrations approaching or exceeding those in the soil under controlled greenhouse conditions (Ruiz and Daniell, 2009). In a related study, Huang et al. (2006) reported using *Chlorella* for mercury bioremediation by expressing the bacterial *merA* gene, which converts Hg^2+^ into Hg^o^, thereby reducing mercury toxicity and promoting its volatilization (Huang et al., 2006). These examples illustrate the potential of engineered organisms, both plants and microalgae, to address mercury pollution through intracellular detoxification.

While these case studies highlight the potential of engineered organisms for mercury bioremediation, they also underscore the need for more comprehensive strategies that address the full spectrum of mercury toxicity. This need has led to the hypothesis of using genetically modified microalgae for intracellular and extracellular detoxification. Unlike surface-displayed MerR systems that merely sequester Hg^2+^ without transformation, our engineered co-expression of MerA and MerB achieves complete detoxification through demethylation and volatilization, reducing bioaccumulation risk while avoiding the biomass disposal problem inherent in adsorption-based approaches.

### 4.3 Hypothesis: extracellular and intracellular detoxification by genetically modified microalgae

While the *mer* operon's function is well-characterized in bacteria, functional equivalence in eukaryotic microalgae cannot be assumed without genomic integration validation and empirical demonstration of enzyme kinetics, cofactor compatibility, and regulatory coupling—key limitations we explicitly acknowledge. Given the limitations of intracellular mercury transformation, such as intracellular toxicity and limited bioconversion efficiency, we propose two innovative approaches: the use of genetically modified microalgae (such as *C. reinhardtii*) to perform mercury detoxification both extracellularly and intracellularly (Figure 1). With the rapid advancements in gene editing and synthetic biology, it is feasible to engineer microalgae to express MerA and MerB either intracellularly or on their cell surface. *C. reinhardtii* offers several advantages, including its large surface area, ability to efficiently contact pollutants in water, and natural capacity to bind mercury (Krishnamoorthy et al., 2025).

For intracellular detoxification, expressing MerA and MerB within the microalgae can be complemented by incorporating mercury transport channels from the *mer* operon, such as mercury transport protein C (MerC), mercury transport protein F (MerF), broad-spectrum mercury transport protein E (MerE), organomercurial transport protein (MerG), mercury transport protein T (MerT), and mercury-binding periplasmic protein (MerP), to enhance the uptake and translocation of mercury across cell membranes (Sone et al., 2013). This strategy aims to enhance the efficiency of mercury biotransformation by facilitating increased mercury uptake into cells, where MerA and MerB would theoretically detoxify it. However, actual uptake rates and intracellular processing capacity in microalgae are unknown. Alternatively, for extracellular detoxification, surface display technology can anchor MerA and MerB on the cell surface of microalgae. This approach is designed to enable the direct conversion of toxic mercury compounds into Hg^o^ in the extracellular environment, potentially reducing their bioavailability before cellular uptake. The transformed Hg^o^ would be released into the atmosphere, which may reduce local aquatic risks. However, this transfer between environmental compartments requires a comprehensive life-cycle assessment to evaluate potential atmospheric re-oxidation and long-range deposition. Projections based on bacterial kinetics suggest that the sequential action of MerB and MerA might achieve near-complete detoxification within minutes to hours under idealized laboratory settings (Priyadarshanee et al., 2022). Bacterial studies have demonstrated the rapid cleavage of carbon-mercury bonds by MerB, followed by Hg^2+^ reduction by MerA (Liu et al., 2025). However, whether volatilization efficiencies exceeding 90% can be attained in microalgal systems remains an untested hypothesis requiring empirical validation.

The underlying volatilization mechanisms warrant explicit discussion, though critical uncertainties remain when extrapolating bacterial paradigms to eukaryotic microalgae. Intracellular Hg^o^ volatilization occurs when MerA reduces Hg^2+^ using NADPH as an electron donor (Yu et al., 2025). In *Chlamydomonas*, we hypothesize that photosynthesis may provide reducing equivalents to sustain MerA activity. However, direct measurements of NADPH flux and MerA turnover rates in algal chloroplasts are currently lacking in the literature. The passive diffusion of lipid-soluble Hg^o^ across thylakoid and plasma membranes, while thermodynamically favorable, has not been quantified in microalgal systems. Extracellular Hg^o^ volatilization via surface-displayed MerA presents additional unresolved challenges: protein anchoring strategies compatible with algal cell walls, enzymatic stability against aquatic proteolysis, and optimal spatial orientation for substrate channeling remain uncharacterized. While bacterial studies suggest that surface-displayed MerB and MerA could minimize diffusion losses, mass transfer limitations at the cell-water interface, Hg^o^ photoreactivity, and potential re-oxidation in surface waters introduce kinetic constraints that require empirical evaluation (Guo and Hui, 2025). We therefore propose specific validation experiments: (1) quantifying *in vivo* NADPH/NADP^+^ ratios and MerA catalytic efficiency in transgenic *C. reinhardtii*; (2) measuring Hg^o^ volatilization rates using gas-phase atomic fluorescence spectrometry; (3) assessing enzyme stability and activity retention in natural water matrices over timescales relevant to bioremediation. These uncertainties regarding the mechanisms need to be resolved before we can have a meaningful discussion about scalability.

Translating bacterial surface-display systems to microalgae involves fundamental engineering challenges that remain unresolved. Unlike Gram-negative bacteria, which have well-characterized outer membrane protein anchors, microalgae possess complex, multilayered cell walls that lack established anchoring motifs (Nakanishi et al., 2022). Potential strategies include fusion to algal cell wall proteins or glycosylphosphatidylinositol anchors, though their efficiency and orientation control are uncharacterized for large heterologous enzymes. Protein stability in aquatic environments presents another critical bottleneck: extracellular MerA/MerB would be exposed to proteases, oxidative stress, and pH fluctuations, which could potentially reduce their functional half-lives. Enzyme orientation must ensure catalytic domains face outward while maintaining membrane integration, and crowding effects from high-density display could impair substrate access. These constraints require experimental validation of anchor selection, proteolytic resistance assays in natural water matrices, and a quantitative comparison of volatilization rates between free and surface-anchored enzymes before this strategy can be deemed feasible.

However, efficiency depends on mass transfer dynamics at the cell-water interface. Microscale turbulence facilitates Hg^o^ desorption, but stagnant conditions can lead to re-oxidation or re-adsorption (Priyadarshanee et al., 2022). Furthermore, Hg^o^ photoreactivity in surface waters introduces a competing process that may decrease net volatilization, requiring optimization of enzyme density and hydrodynamic conditions. By leveraging the natural capabilities of microalgae, this dual strategy represents a conceptual framework that, pending experimental validation, could evolve into a sustainable approach for mercury bioremediation, with the long-term goal of addressing both organic and inorganic forms of mercury.

This volatilization strategy exemplifies the central trade-off discussed below. While Hg^o^ formation reduces the risk of aquatic methylmercury bioaccumulation, it introduces atmospheric transport pathways that must be weighed against the benefits of *in-situ* remediation through quantitative mass balance analyses.

### 4.4 Critical considerations and challenges for GMO-based mercury bioremediation

The release of genetically modified microalgae into open aquatic ecosystems for mercury detoxification presents several practical challenges and biosafety concerns that must be carefully evaluated, including genetic stability, horizontal gene transfer (HGT) to indigenous microbes, and potential disruption of native mercury-cycling communities. While some studies suggest a limited risk of HGT in controlled settings (Caliendo et al., 2025), others argue that current biosafety models inadequately capture rare transfer events in complex aquatic microbial communities, potentially underestimating the long-term ecological impacts (Eckerstorfer et al., 2025; Moon, 2025). Despite the transformative potential of engineered microalgae, the critical challenges above must be addressed prior to field deployment. Genetic stability represents a primary concern: plasmid-borne *mer* operons may be lost in non-selective environments, while constitutive expression imposes metabolic burdens that reduce strain fitness over generations. Chromosomal integration and stress-responsive promoters, as demonstrated in bacterial biosensors, offer potential solutions but require validation in open aquatic systems (Hui et al., 2023a; Caliendo et al., 2025).

Biosafety and ecological risks pose equally significant hurdles. The HGT of engineered mercury components to indigenous microbes remains poorly quantified, and the volatilization of Hg^o^—although reducing aquatic bioavailability—transfers mercury to the atmosphere. While Hg^o^ volatilization reduces aquatic toxicity, it transfers mercury to the atmosphere, where long-range transport and subsequent re-oxidation can redeposit toxic forms in remote ecosystems, necessitating a comprehensive life-cycle assessment of the overall environmental impact.

The indiscriminate release of GMOs could disrupt native mercury-cycling communities, altering methylation-demethylation equilibria in unpredictable ways (Bisson, 2025). Surface-display strategies reduce intracellular accumulation but do not remove the risks of cell lysis and the release of heavy metals into the environment (Guo and Hui, 2025).

Regulatory frameworks and biomass disposal present additional barriers. Existing GMO biosafety protocols inadequately address aquatic microbial biocontrol agents, and harvesting free-floating microalgae remains a technically challenging task. Field-scale deployment of engineered microalgae faces significant regulatory and ethical constraints, including a lack of specific biosafety protocols for aquatic GMOs, requirements for long-term ecological monitoring, and public concerns about irreversible environmental release of synthetic organisms. Public perception of the environmental release of GMOs makes transparent risk-benefit communication essential (Moon, 2025). These factors underscore the need for technological innovation to be coupled with thorough ecological risk assessments and adaptive governance.

### 4.5 Future perspectives: advanced technologies for next-generation bioremediation

Emerging technologies offer pathways to overcome these challenges. CRISPR/Cas-based genome editing enables precise, markerless integration of *mer* operons into microalgal genomes, enhancing stability and reducing metabolic burden. Recent advances in algal editing enable the fine-tuning of MerA/MerB expression to optimize detoxification efficiency while maintaining cellular fitness (Feng et al., 2023).

Synthetic biology-enabled circuits can program microalgae with Hg-responsive modules that activate only upon exposure, minimizing metabolic drain. Logic gates and toggle switches, which improved bacterial biosensor specificity, could synchronize detoxification across algal populations via quorum sensing (Liu et al., 2022). Integrating recovery and biosecurity systems—such as Hg-activated fluorescent markers for harvesting or environmental cue-triggered suicide modules—could address containment concerns (Xue et al., 2022).

Omics technologies will accelerate strain development. Comparative genomics of diverse mercury-resistant bacteria can identify superior *mer* operon variants, while proteomics and metabolomics can guide rational chaperone co-expression to protect MerA/MerB from oxidative damage. Machine learning, already applied to optimize heavy metal biosensors, could predict optimal *mer* transporter combinations for enhanced MeHg uptake (Sharma et al., 2025).

Furthermore, immobilization strategies offer a promising avenue for enhancing the practical viability of engineered microalgae. Immobilized algae systems exhibit increased biosorption capacity compared to free cells, as they prevent biomass loss during treatment cycles (Jiang et al., 2023). Integrating immobilization platforms with genetically engineered microalgae, such as carriers for surface-displayed MerA/MerB or as pre-concentration matrices, could effectively address the critical challenge of biomass harvesting while enhancing process stability and detoxification efficiency.

Looking forward, synthetic microbial consortia—combining mercury-detoxifying microalgae with bacterial partners—may offer a hybrid solution. Such ecosystems could leverage bacterial methylation/demethylation activities while microalgae provide photosynthetic niches and facilitate Hg^o^ volatilization, creating a self-regulating platform that mimics natural biogeochemical cycles with enhanced safety controls. To validate this conceptual framework, future work will prioritize the laboratory-scale characterization of engineered microalgae strains, coupled with mesocosm studies and computational modeling of mercury flux dynamics, to optimize enzyme expression levels and assess long-term ecological impacts before conducting pilot-scale field trials.

## 5 Conclusion

This opinion article highlights a critical limitation in current mercury bioremediation research—the predominant focus on inorganic Hg^2+^ while overlooking the more toxic MeHg. We propose transformative strategies that utilize genetically modified microalgae capable of both intracellular and extracellular detoxification, with the ultimate goal of developing systems that can address both forms of mercury, pending laboratory-scale validation and biosafety assessments. While challenges, including genetic stability, biosafety concerns, and regulatory limitations, remain significant barriers, advancements in CRISPR/Cas gene editing, synthetic biology circuits, and omics technologies offer promising solutions. Developing next-generation microbial strains with enhanced mercury tolerance and recovery efficiency represents a crucial step toward the comprehensive eco-restoration of contaminated aquatic ecosystems and the protection of human health.
